# Integrated Analysis of Nine Prognostic RNA-Binding Proteins in Soft Tissue Sarcoma

**DOI:** 10.3389/fonc.2021.633024

**Published:** 2021-05-07

**Authors:** Lu-Lu Lin, Zi-Zhen Liu, Jing-Zhuo Tian, Xiao Zhang, Yan Zhang, Min Yang, Hou-Cheng Zhong, Wei Fang, Ren-Xiong Wei, Chao Hu

**Affiliations:** ^1^ Department of Pathology and Pathophysiology, School of Basic Medicine, Wuhan University, Wuhan, China; ^2^ The Third Clinical School, Hubei University of Medicine, Shiyan, China; ^3^ Department of Hepatobiliary and Pancreatic Surgery, Zhongnan Hospital of Wuhan University, Wuhan, China; ^4^ Department of Spine and Orthopedic Oncology, Zhongnan Hospital of Wuhan University, Wuhan, China; ^5^ Hubei University of Medicine, Shiyan, China

**Keywords:** soft tissue sarcoma, RNA binding proteins, biomarker, prognostic model, nomogram

## Abstract

RNA-binding proteins (RBPs) have been shown to be dysregulated in cancer transcription and translation, but few studies have investigated their mechanism of action in soft tissue sarcoma (STS). Here, The Cancer Genome Atlas (TCGA) and Genotype-Tissue Expression (GTEx) databases were used to identify differentially expressed RBPs in STS and normal tissues. Through a series of biological information analyses, 329 differentially expressed RBPs were identified. Functional enrichment analysis showed that differentially expressed RBPs were mainly involved in RNA transport, RNA splicing, mRNA monitoring pathways, ribosome biogenesis and translation regulation. Through Cox regression analyses, 9 RBPs (BYSL, IGF2BP3, DNMT3B, TERT, CD3EAP, SRSF12, TLR7, TRIM21 and MEX3A) were all up-regulated in STS as prognosis-related genes, and a prognostic model was established. The model calculated a risk score based on the expression of 9 hub RBPs. The risk score could be used for risk stratification of patients and had a high prognostic value based on the receiver operating characteristic (ROC) curve. We also established a nomogram containing risk scores and 9 key RBPs to predict the 1-year, 3-year, and 5-year survival rates of patients in STS. Afterwards, methylation analysis showed significant changes in the methylation degree of BYSL, CD3EAP and MEX2A. Furthermore, the expression of 9 hub RBPs was closely related to immune infiltration rather than tumor purity. Based on the above studies, these findings may provide new insights into the pathogenesis of STS and will provide candidate biomarkers for the prognosis of STS.

## Introduction

Soft tissue sarcoma (STS) accounts for less than 1% of all cancers but is highly heterogeneous in terms of anatomical location, histology, molecular characteristics, and prognosis (1). STS originates from mesenchyme and can occur anywhere in the body, with approximately 50% to 60% of cases happening in the extremities (2). In the past few decades, through the use of multidisciplinary methods to control diseases, including surgery, radiation therapy and systemic therapy, the treatment effect among STS patients has greatly improved (3). However, due to its easy metastasis and recurrence, the prognosis of advanced patients is still poor. At present, the diagnosis of STS mainly depends on ultrasound scanning, imaging examination and tissue biopsy, which have difficulty meeting the clinical requirements (4). To reduce the recurrence rate and mortality, enhance quality of life and improve survival rates in STS patients, early detection, diagnosis, and treatment are essential. Over the years, molecular research has shown excellent results in understanding cellular and molecular mechanisms that drive tumorigenesis and progression (5). Therefore, it is necessary to find genes for the occurrence, development and prognosis of STS to further broaden the research.

RNA binding proteins (RBPs) are involved in various RNA metabolism and multiple biological processes, and play an important role in regulating RNA stability, splicing, modification, localization and protein translation (6, 7). RBPs are mainly involved in posttranscriptional control (8), and their disorders are closely related to the occurrence and development of cancer (9–11). There are numerous human RBPs, but the role of RBPs in cancer is not fully known. Only a few experiments have systematically analyzed the RBPs associated with cancer. For example, RBMS2 plays a role in suppressing cancer in breast cancer and actively regulates the expression of P21 by stabilizing its mRNA (12). The overexpression of ESRP1 in ovarian cancer promotes the transformation of ovarian cancer cells from mesenchymal phenotype to epithelial phenotype (13). In addition, some studies focusing on the comprehensive analysis of RBPs found that DCAF13, EZR, MRPL13, APOBEC3C and EIF4E3 may have a prognostic value in breast cancer (14). It was reported that PUM2 could partly and competitively bind to STARD13 3’UTR with miRNAs to achieve the effect of inhibiting osteosarcoma progression (15). RBM10 was also demonstrated to be tumor suppressor in osteosarcoma (16). However, few studies have investigated the different roles of RBPs in STS. Therefore, based on the above evidence, RBPs may also have a regulatory effect on STS. This provides a new direction for our study of STS.

The methylation and the resulting abnormal expression of target gene play an important role in the tumorigenesis in multiple tumors including STS (17, 18). It was reported that the methylation status was also associated with prognosis in STS (19). Furthermore, immune infiltration has the vital impact on tumor biological behaviors through activating or suppressing innate and adaptive immunity (20, 21). Hence, in this study, we investigated the relationship between STS and RBPs using bioinformatics methods to better investigate the pathological mechanisms of RBP in STS. We also analyzed the signaling pathways, potential functions, prognostic value, gene alterations, methylation levels and immune infiltration of RBPs in STS. These results not only helped us discover new genes related to RBPs for diagnosis but also increased our understanding of STS to further define prognosis and screen new drug targets to improve the treatment of STS.

## Materials and Methods

### Data Collection and Processing

Corresponding gene expression profiles and clinical data were obtained from The Cancer Genome Atlas database (TCGA) and Genotype-Tissue Expression (GTEx) database using the University of California at Santa Cruz (UCSC, https://genome.ucsc.edu) Genome Website. RNA-sequencing data (FPKM values) of STS in TCGA-SARC cohort (https://xenabrowser.net/datapages/?cohort=GDC%20TCGA%20Sarcoma%20(SARC)&removeHub=https%3A%2F%2Fxena.treehouse.gi.ucsc.edu%3A443) and normal fat tissue samples in GTEx database (https://xenabrowser.net/datapages/?cohort=GTEX&removeHub=https%3A%2F%2Fxena.treehouse.gi.ucsc.edu%3A443) were included in this study. After downloading gene expression datasets, ENSEMBLE identifiers were matched to official gene symbols. We created a unified processing before the two sets of data were merged for analysis. Because the obtained STS gene expression profiles have been transformed by log2(x+1), the normal fat tissue gene expression data were also manipulated by using log2(x+1) transformation. Then, two gene files were merged and standardized, and the expression matrix of RBPs was extracted for differential analysis. Ultimately, differentially expressed RBPs were identified based on |logfold change (FC)| > 0.5 and false discovery rate (FDR) < 0.05, using Wilcoxon test in R software. Wilcoxon test is one of the most common nonparametric test methods (22).

### Functional Enrichment Analyses

Gene Ontology (GO) and Kyoto Encyclopedia of Genes and Genomes (KEGG) analyses were performed to find the biological functions of these differentially expressed RBPs. GO categories were comprised of three terms: molecular function (MF), biological process (BP), and cellular component (CC). KEGG analysis was used to find the key pathways that these genes were involved in STS. All enrichment analyses were performed based on background genes from GO or KEGG database using the “clusterProfiler” package (23) in R software. Both P and FDR values less than 0.05 were the threshold for the identification of significant GO terms and KEGG pathways.

### Protein–Protein Interaction (PPI) Network Construction and Module Screening

Differentially expressed RBPs were uploaded to the STRING database (https://srting-db.org/cgi/input.pl) (24) to obtain a protein-protein interaction (PPI) network. Subsequently, Cytoscape3.7.0 software was used to study PPI network. We used Centiscape 2.2 to obtain centrality indexes of nodes of network. Molecular Complex Detection (MCODE) (25) was used to find and visualize the top 3 clusters of the PPI network. Selection criteria were as follows: degree cutoff = 2, node score cutoff = 0.2, K - Core = 2. After that, the shared genes between the PPI network and the differentially expressed RBPs were extracted for subsequent model construction.

### Prognostic Model Construction

All samples were randomly divided into 2 groups (training set and test set) with the ratio of 1:1. The training group was used to construct a prognostic model, while the test group was used to verify the result of the training group. Since there was little clinical information in the training and testing groups, we also validated the prognostic model in the whole population. First, univariate Cox regression analysis of all samples was performed to screen differentially expressed RBPs (P < 0.01 was considered statistically significant). Then, multivariate Cox regression analysis was performed to find the optimal genes to construct a prognostic model in the training group. These optimal genes were called as hub genes. Risk score for STS patients was calculated based on the following formula: risk score = $\Sigma_{i=1}^{n}(\beta i*Expi);$ where i represented each gene, n represented the total number of hub genes in the multivariate Cox regression analysis, βi represented gene coefficient value and Expi represented the expression value of each gene. According to the median risk score, STS patients were divided into a high-risk group and a low-risk group. The Kaplan-Meier survival curve was drawn to compare the survival difference between the two groups in the training set. In addition, we used the “survivalROC” package to draw a receiver operation curve (ROC) (26) to evaluate the predictive ability of the prognostic model. To further validate our results, the prognostic model was also used to calculate the risk score for each patient in the test group and the whole cohort. Similarly, Kaplan-Meier survival curve and the ROC curve were plotted to evaluate the predictive ability of the model in both the validation and whole cohorts. P < 0.05 was considered statistically significant for survival differences between the high and low-risk groups.

### Identification and Analysis of Independent Prognostic Factors

Univariate and multivariate Cox regression analyses were used to evaluate the effect of multiple clinical factors (age, sex, metastasis, radiotherapy, and risk score) on the prognosis of STS patients to examine the accuracy of the STS prognostic model and determine independent prognostic factors (P < 0.05 in both analyses). Finally, we used the “rms” package in the R software to construct a nomogram to accurately predict each patient’s survival rate.

### Alterations of Hub RBPs

Rates of hub RBP changes in STS were carried out by querying the online cBioPortal (http://cbioportal.org/), which provides complex tumor genomics (27). Through this website, we explored the impact of hub RBP changes on the survival of STS.

### Methylation Analyses

Human Disease Methylation Database Version 2.0 (DiseaseMeth2.0 (http://biobigdata.hrbmu.edu.cn/diseasemeth/) was used to compare the methylation degree of hub RBPs between STS and normal tissue. This database collects and analyzes DNA methylation data in various human diseases (28, 29). In addition, we also used MEXPRESS (http://mexpress.be) (30) to study the relationship between hub RBPs and their methylation status.

### Immune Infiltration Analysis

There is evidence that tumor cell immune infiltration is closely related to prognosis (31). For example, in LUSC, T follicular helper cells are associated with a good prognosis, and an increase in neutrophils indicates a poor prognosis (32). Tumor immune estimation resource (TIMER) is a comprehensive resource for systematic analysis of various malignant tumors. TIMER contains molecular characterization of 32 types of cancer and 6 types of immune cells (33). Based on the TIMER database, we further evaluated the relationship between immune cell types (CD4+T cells, CD8+T cells, B cells, dendritic cells, macrophages, neutrophils and tumor purity) and 9 hub RBPs in STS.

### Potential Interactions of Hub RBPs

RNA Interactome Database (RNAInter) (http://www.rna-society.org/rnainter/ or http://www.rna-society.org/raid/.) (34) is a platform to investigate RNA associated interactions, which involve many physiological and pathological processes. We used RNAInter to explore the potential targets of 9 hub RBPs and RBPs-associated interactions to facilitate the understanding of 9 hub RBPs and further research of RBPs on STS.

## Results

### The Identification of RBPs in STS Patients

**Figure 1:**
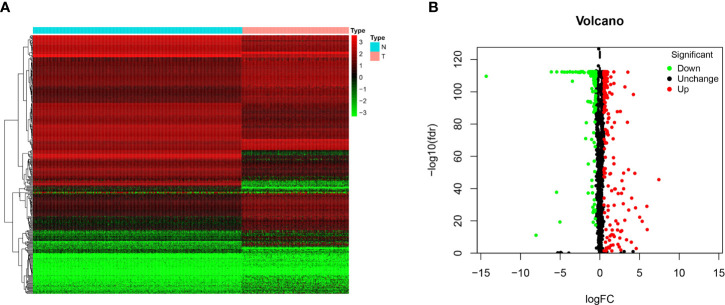
The differentially expressed RBPs in STS. **(A)** Heat map of 329 differentially expressed RBPs between tumor (T) and normal fat tissue (N) samples based on FDR < 0.05 and |logFC|>0.5; **(B)** Volcano plot of all RBPs. The red dots represent up-regulated RBPs. The green dots represent down-regulated RBPs. The black dots represent RBPs with no significant changes between tumor (T) and normal fat tissue (N) samples based on FDR < 0.05 and |logFC|>0.5.

In this study, 263 tumor samples and 517 normal tissue samples were downloaded from TCGA and GTEx databases, respectively. Only 259 patients had survival data. There were seven histological types in 259 patients and specific information was presented in **Supplementary File 1**. A total of 1492 RBPs were extracted for the identification of differentially expressed RBPs between the STS group and normal fat tissue group (**Supplementary File 2**). According to the screening criteria (FDR < 0.05, |logFC|>0.5), 329 RBPs were obtained in this study, including 191 upregulated and 138 downregulated (**Figure 1**). The detailed information of 329 RBPs was summarized in **Supplementary File 3**.

### GO Terms and KEGG Pathway Analysis of the Differentially Expressed RBPs

**Figure 2:**
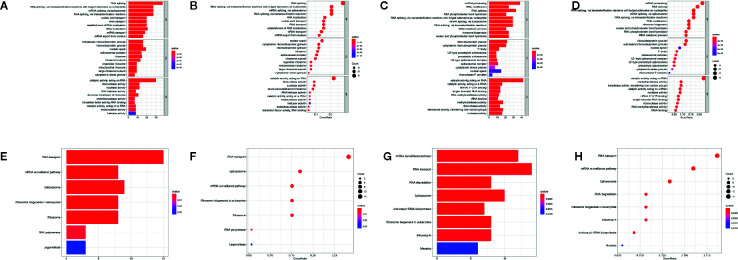
Functional enrichment analyses for differentially expressed genes. GO enrichment analysis for down-regulated RBPs **(A, B)** and up-regulated RBPs **(C, D)**. KEGG pathways enrichment for down-regulated RBPs **(E, F)** and up-regulated RBPs **(G, H)**.

To elucidate the potential biological functions and mechanisms related to the differentially expressed RBPs, GO and KEGG functional analyses were performed. In GO analysis, downregulated genes were enriched in RNA splicing, nucleic acid transport, RNA localization, cytoplasmic ribonucleoprotein granule, nuclear speck, and ribonuclease activity (**Figures 2A, B**), and upregulated genes were enriched in ncRNA processing, RNA modification, tRNA metabolic process, ribonucleoprotein granule, mRNA 3’−UTR binding, and methyltransferase activity (**Figures 2C, D**). In addition, KEGG analysis of these genes showed that they were mainly involved in the RNA transport, mRNA surveillance pathway, spliceosome and ribosome biogenesis in eukaryotes (**Figures 2E–H**).

### Protein-Protein Interaction (PPI) Network Construction and Key Module Selection

**Figure 3:**
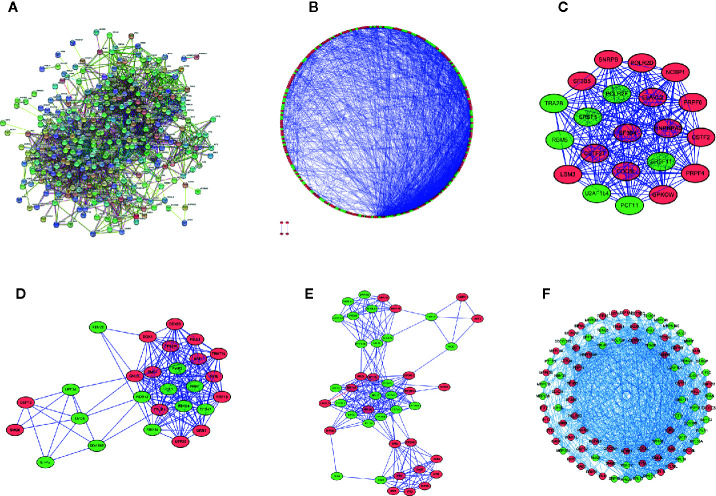
Protein-protein interaction network and module visualization. **(A)** Initial protein-protein interaction analysis of 329 differentially expressed RBPs. **(B)** the visualization of Protein-protein interaction network of 329 differentially expressed RBPs. Critical module 1 **(C)**, 2 **(D)**, and 3 **(E)** from PPI network using MCODE in Cytoscape. Module 1 contains 21 points and 209 edges, Module 2 contains 27 points and 190 edges, and module 3 contains 44 points and 236 edges. **(F)** Visualization of the integration of module 1,2 and 3. Green circles represent down-regulated RBPs, red circles represent up-regulated RBPs.

To explore the interaction relationship between the differentially expressed RBPs, we constructed a PPI network containing 2110 edges in the STRING database (**Figure 3A**, **Supplementary File 4**). These results were further analyzed and visualized by Cytoscape, and we obtained a PPI network with 302 nodes and 2075 edges (**Figure 3B**). **Supplementary File 5** showed centrality indexes of nodes in PPI network. To explore the hub RBPs in STS, the PPI network was analyzed by MCODE in Cytoscape, and three important modules were selected (**Figures 3C–E** and **Supplementary File 6**). Module 1 contained 21 points and 209 edges, Module 2 contained 27 points and 190 edges, and module 3 contained 44 points and 236 edges (**Figures 3C–E** and **Supplementary File 6**). The genes from three modules were visualized in a network (**Figure 3F**). Finally, 272 shared genes (**Supplementary File 3**) between the PPI network and 329 differentially expressed RBPs were extracted for subsequent analyses.

### Prognosis-Related RBP Selection

**Figure 4:**
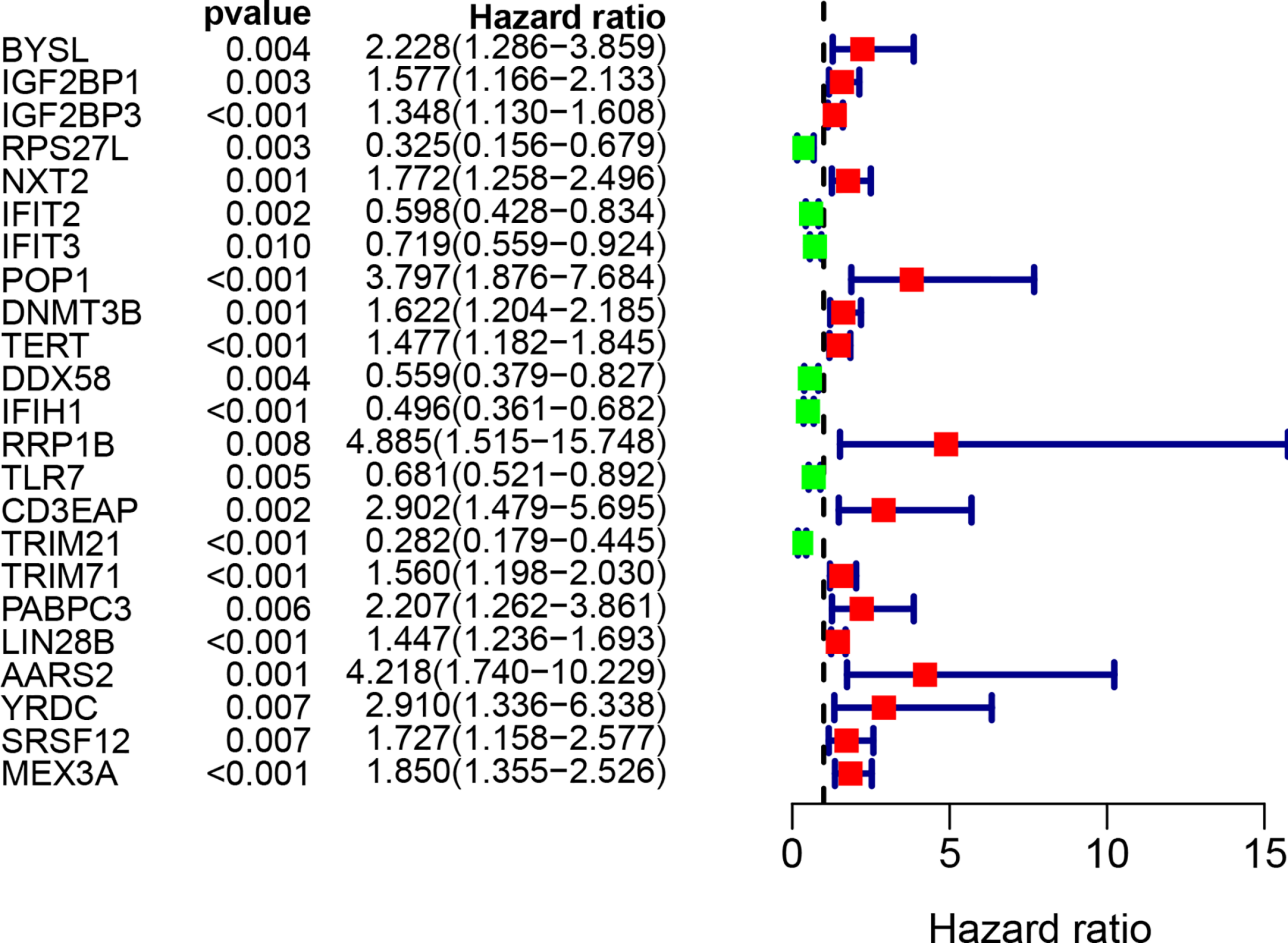
Univariate Cox regression analysis for identification of survival related RBPs. The forest plot of survival related RBPs based on univariate Cox regression analysis (P < 0.01). Red square, hazard ratio > 1; Green square, hazard ratio < 1.

**Figure 5:**
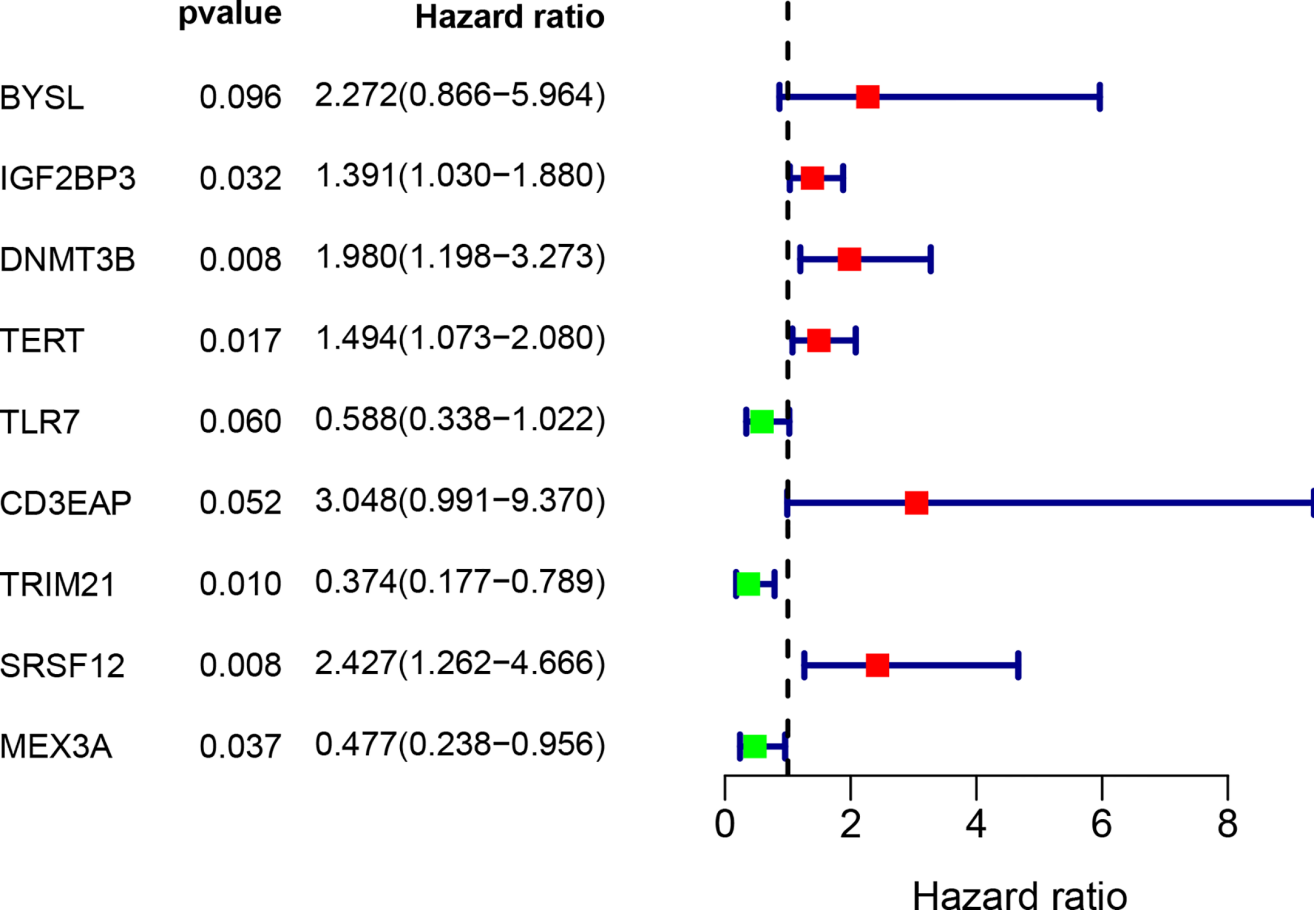
Multivariate Cox regression analysis to identify prognosis related 9 hub RBPs. The forest plot of 9 hub RBPs based on multivariate Cox regression analysis. Red square, hazard ratio > 1; Green square, hazard ratio < 1.

By combining gene expression and survival information, 259 patients were used for subsequent model construction. The 259 patients were randomly divided into two groups: training set (N = 131) and test set (N = 128). To select RBPs associated with overall survival (OS), univariate Cox regression analysis was performed on 272 RBPs in 259 patients. The results showed that 23 differentially expressed RBPs were significantly correlated with OS (P < 0.01, **Figure 4**). Then, the above 23 RBPs were used for further multivariate Cox regression analysis, and finally 9 RBPs highly correlated with STS survival were obtained to establish a prognostic model in the training group (**Figure 5**).

### Construction and Evaluation of a Prognostic Model Associated With RBPs

We obtained 9 RBPs to calculate a risk score and construct a prognostic model. The patient Risk Assessment formula was as follows (**Table 1**):

$$\begin{array}{l} \text{Risk score}=(0.821*\text{Exp}(\text{BYSL}))+(0.330*\text{Exp}(\text{IGF}2\text{BP}3))+(0.683*\text{Exp}(\text{DNMT}3\text{B}))+(0.402*\text{Exp}(\text{TERT}))-(0.531*\text{Exp}(\text{TLR}7))\\ \;+(1.114*\text{Exp}(\text{CD}3\text{EAP}))-(0.983*\text{Exp}(\text{TRIM}21))+(0.887*\text{Exp}(\text{SRSF}12))-(0.740*\text{Exp}(\text{MEX}3\text{A})) \end{array}$$

**Table 1:** Nine prognosis-associated hub RBPs identified by multivariate Cox regression analysis.

Genes	Overall survival				
	coef	HR	95% CI		p value
**BYSL**	0.821	2.272	0.866	5.964	0.096
**IGF2BP3**	0.330	1.391	1.030	1.880	0.032
**DNMT3B**	0.683	1.980	1.198	3.273	0.008
**TERT**	0.402	1.494	1.073	2.080	0.017
**TLR7**	-0.531	0.588	0.338	1.022	0.060
**CD3EAP**	1.114	3.048	0.991	9.370	0.052
**TRIM21**	-0.983	0.374	0.177	0.789	0.010
**SRSF12**	0.887	2.427	1.262	4.666	0.008
**MEX3A**	-0.740	0.477	0.238	0.956	0.037

coef, coefficient; HR, hazard ratio; CI, confidence interval.

**Figure 6:**
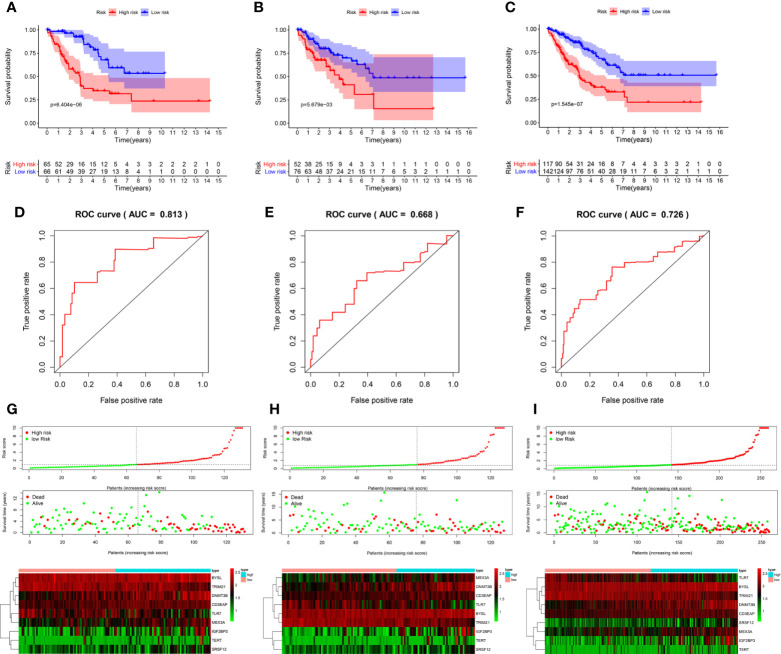
Evaluation of the prognostic model. Survival analyses for the training **(A)**, test **(B)**, and overall (training + test, **C**) cohorts. Receiver operating characteristic (ROC) curves of the prognostic model in the training **(D)**, test **(E)**, and overall **(F)** cohorts. Distribution of risk score, survival time, and expression heat map between the high (red dots)- and low(green dots)-risk groups in the training **(G)**, test **(H)**, and overall **(I)** cohorts. AUC: area under the curve.

According to the median risk score, 131 patients in the training group from TCGA database were divided into the high-risk group and the low-risk group. Survival analysis showed that high-risk patients had shorter OS (P = 6.404e-06, **Figure 6A**). Next, the area under the ROC curve (AUC) was 0.813, indicating that this model had high predictive ability in the training group (**Figure 6D**). Similarly, the risk score could also predict the survival rate (P = 5.679e-03 and P = 1.545e-07, respectively; **Figures 6B, C**) of patients and had an accurate predictive ability (AUC = 0.668 and AUC = 0.726, respectively; **Figures 6E, F**) in the test group and the whole cohort, respectively. These results suggested that the risk score can predict patient survival well, and the model had good sensitivity and specificity. Moreover, the expression of hub RBPs was also significantly different between high- and low-risk groups (**Figures 6G–I**).

### Identification of Independent Prognostic Factors and Construction of the Nomogram

**Figure 7:**
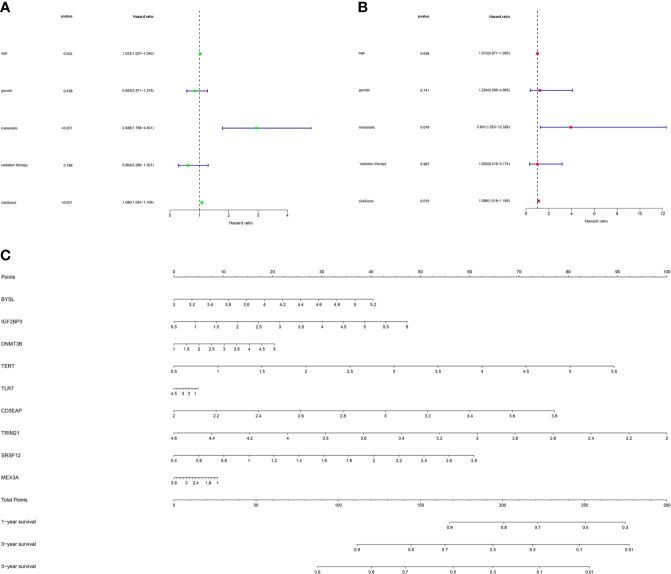
The prognostic value of different clinical factors and nomogram construction. **(A)** Univariate analysis of different clinical factors of STS. **(B)** Multivariate analysis of different clinical factors of STS. **(C)** A nomogram containing the expression of 9 RBPs and risk score for the prediction of 1-, 3-, and 5-year overall survival in STS patients.

As shown in **Figure 7A**, the univariate Cox regression analysis showed that factors including age, metastasis and risk score affected the survival of STS patients (P < 0.05). Subsequently, multivariate Cox regression analysis further demonstrated that metastases and risk scores were independent prognostic factors (P < 0.05, **Figure 7B**). Based on the above regression analyses, the risk score results further confirmed the accuracy of the prognostic model. Finally, we constructed a nomogram to predict 1-year, 3-year, and 5-year survival rates of patients (**Figure 7C**).

### Relationship Between Hub RBP Alterations and OS

**Figure 8:**
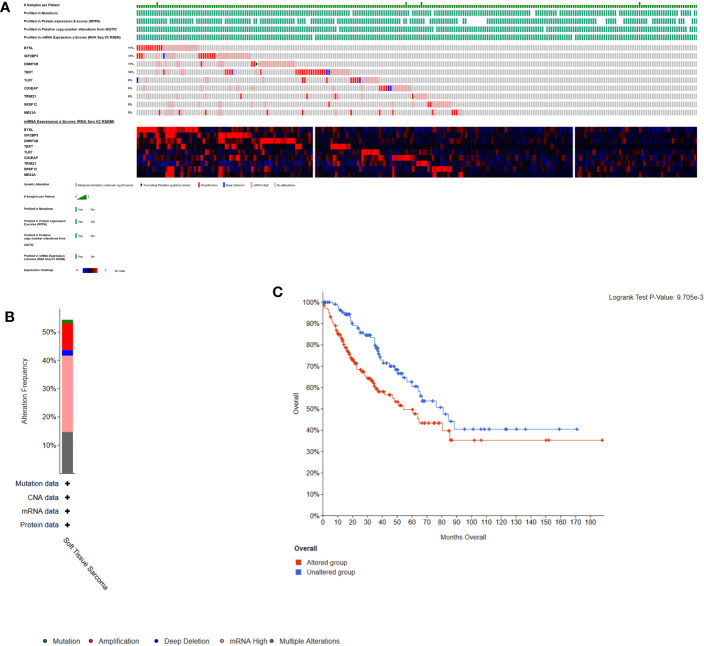
Alterations in expression of the 9 hub RBPs. **(A)** A total of 148 of 261 samples (57%) had alterations of the 9 hub RBPs based on data about mutations, protein, copy-number alterations and mRNA expression. Missense Mutation(unknown significance): samples had missense mutations of RBPs with unknown biological significance. Truncating Mutation(putative driver): driver mutation. mRNA High: samples had high mRNA expression of RBPs. **(B)** Frequencies of different alterations. **(C)** Survival analysis for patients with and without alterations in the 9 hub RBPs. Unaltered group had better survival.

Here, cBioPortal was used to study the hub RBPs in 261 STS samples. **Figures 8A, B** showed that alterations of hub RBPs were found in 57% (148/261) of the patients. Alterations in these RBPs led to a poor prognosis (P < 0.05, **Figure 8C**).

### Methylation Analysis of Key RBPs

**Figure 9:**
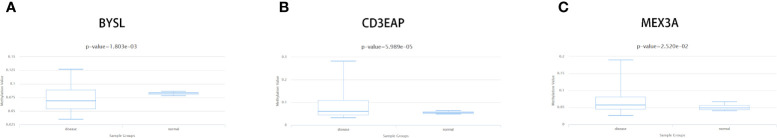
Methylation analyses of STS hub RBPs. The significant changes of methylation level about **(A)** BYSL, **(B)** DD3EAP, and **(C)** MEX3A between STS and controls.

In this study, we used the DiseaseMeth version 2.0 to further analyze the methylation of these 9 differentially expressed RBPs. **Figures 9A–C** showed significant differences in methylation of BYSL (P = 1.803e-03), CD3EAP (P = 5.989e-05) and MEX3A (P = 2.520e-02). Furthermore, using MEXPRESS analysis, we observed that multiple methylation sites in the RBP sequence were negatively correlated with their own expression levels (P < 0.05, **Supplementary Figure 1**).

### Hub RBP Immune Infiltration Analysis

**Figure 10:**
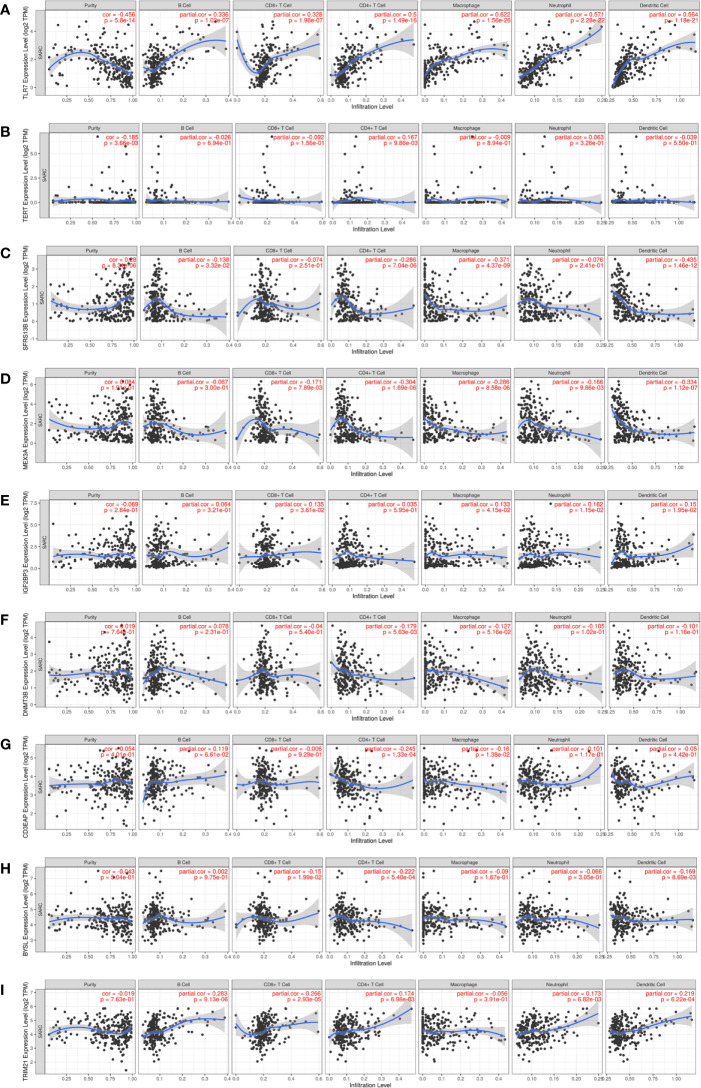
Relationships between expression of 9 hub RBPs and tumor immune infiltrations. **(A)** TLR7, **(B)** TERT, **(C)** SRSF12, **(D)** MEX3A, **(E)** IGF2BP3, **(F)** DNMT3B, **(G)** CD3EAP, **(H)** BYSL and **(I)** TRIM21.

Given that immune cells are involved in the composition of the tumor microenvironment, which is of great significance to the prognosis of the tumor, we studied the potential connection between 9 hub RBPs and immune infiltration (purity, B cell, CD8+T cell, CD4+T cell, macrophage, neutrophil, dendritic cell) in the STS. **Figure 10A** showed that TLR7 was positively correlated with B cells (partial. cor = 0.336, P = 1.02e-07), CD8+ T cells (partial. cor = 0.328, P = 1.98e-07), CD4+ T cells (partial. cor = 0.5, P = 1.49e-16), macrophages (partial. cor = 0.622, P = 1.56e-26), neutrophils (partial. cor = 0.571, P = 2.28e-22) and dendritic cells (partial. cor = 0.564, P = 1.18e-21). Similarly, TRIM21 and IGF2BP3 were also positively correlated with most immune cells (**Figures 10I, E**). BYSL and MEX3A were negatively associated with most immune cells (**Figures 10H, D**). **Figures 10A–C** indicated that tumor purity was only significantly correlated with TLR7 (cor = -0.456, P = 5.8e-14), TERT (cor=-0.185, P = 3.66e-03) and SRSF12 (cor = 0.28, P = 8.31e-06). In addition, we found that DNMT3B (partial. cor = -0.179, P = 5.63e-03) and CD3EAP (partial. cor = -0.245, P = 1.33e-04) were only negatively associated with CD4+ T cells (**Figures 10F, G**).

### Identification of Potential Interactions of Hub RBPs

We displayed potential interactions of RBPs using “Exact Search” function of RNAInter. **Supplementary Figure 2** showed the top 100 potential interactions of RBPs. BYSL, CD3EAP had more interactions with transcription factors and RBPs. DNMT3B, IGF2BP3, MEX3A, SRSF12 and ILR7 had more interactions with microRNA and RBPs. TERT and TRIM21 had more interactions with microRNA, transcription factors and RBPs.

## Discussion

Currently, accurate diagnosis and prediction of biological behavior have become major challenges because of the STS rarity and complexity. In the course of tumor development, molecular and cellular components are considered to be potential prognostic factors (35). Therefore, finding new treatment targets for STS and elucidating their mechanisms can improve patient prognosis. Recently, bioinformatics methods, such as machine learning and weighted gene co-expression network analysis, have been used in the identification of diagnostic or prognostic biomarkers, as well as molecular subtypes to better understand STS (36, 37). Some of biomarkers have also been validated by basic experiments. Nevertheless, further research is needed on STS. In addition, there were numerous studies about RBPs in cancers (10, 38, 39), but few studies of RBPs in STS. In this study, TCGA and GTEx databases were used to identify potential prognostic genes associated with RBPs to explore the pathways and functions of the RBPs involved in STS. First, we obtained 329 differentially expressed RBPs. Among them, CDC5L (40) and IGF2BP1 (41) have been indicated to be biomarkers of osteosarcoma and affect the prognosis of osteosarcoma. To elucidate the underlying biomolecular mechanism of differentially expressed RBPs, we used GO and KEGG enrichment analyses. Then, the PPI network was constructed by STRING and visualized by Cytoscape. Finally, through univariate and multivariate Cox regression analyses, we obtained 9 RBPs and a prognostic model with high prognostic value. To further explore the role of these 9 RBPs in STS, we further performed the alternation, methylation degree, immune infiltration analyses and identified potential interactions of RBPs. These findings may help to identify RBPs associated with STS and be used for the diagnosis and treatment of STS.

At present, the research of RBPs mainly focuses on posttranscriptional events, which are extremely related to the occurrence of tumor. In this study, GO biological process analysis showed that the differentially expressed RBPs mainly focused on RNA splicing, ribonucleoprotein granule, cytoplasmic ribonucleoprotein granule and nuclease activity. Similar to previous studies, these pathways affected the progression of human diseases (11, 42, 43). RNA splicing is a very important biological process in the human body, and splicing dysregulation is involved in the pathogenesis of various cancers (44). This mechanism has been reported to affect the prognosis and biological behavior of STS (45). RNA modification is a dynamic regulatory process, mainly involved in biological processes such as cell differentiation, and its dysregulation is one of the causes of cancer (46). Translation disorder is common in cancer (47). Both carcinogens and tumor suppressor factors affect the mechanism of translation, making translation abnormalities common in cancer. In addition, KEGG analysis revealed mRNA monitoring pathways, ribosomal biogenesis, and ribosomal lysis pathways that have been reported to be associated with cancer. Previous studies have suggested that ribosomal proteins affect tumor development by regulating the P53 pathway and mRNA translation (48, 49). Overall, GO and KEGG analyses suggested that these RBPs were closely related to the progress of STS through the abovementioned pathways.

To identify the key RBPs, 272 shared genes were extracted from the PPI network and 329 differentially expressed RBPs for further analysis. Then, 9 key RBPs (BYSL, CD3EP, DNMT3B, IGF2BP3, MAX3A, SRSF12, TERT, TLR7 and TRIM21) were obtained using univariate and multivariate Cox regression analyses. They have been reported to participate in the progression and play a key role in the prognosis of different cancers, such as lung cancer, hepatocellular carcinoma, breast cancer, rhabdomyosarcoma, glioma, etc. BYSL is a nucleolar protein involved in the biogenesis of ribosomes through 18s rRNA processing in mammals to affect cell proliferation (50). It is positively expressed in hepatocellular carcinoma (51), prostate cancer (52) and ovarian cancer (53). CD3EAP (also known as ASE1, CAST, ERCC1 antisense) is a component of the RNA polymerase I complex, which is involved in ribosome biosynthesis and promotes cell proliferation similar to BYSL (54). CD3EAP has been reported to be involved in the occurrence and metastasis of lung cancer tissues (55). Although BYSL and CD3EAP play important roles in human cancers, their specific mechanisms in STS remain unclear and need further exploration. DNMT3B, a DNA methyltransferase responsible for *de novo* methylation during human development (56), was significantly elevated in osteosarcoma and rhabdomyosarcoma (57, 58). IGF2BP3 is an embryonic protein that belongs to the mRNA binding protein family and is considered an oncogene (59). It is re-expressed in some cancers and promotes the invasion and migration of tumor cells. In Ewing sarcoma, IGF2BP3 serves as an effective indicator of poor prognosis and predicts the recurrence of patients (60). TERT is a telomerase reverse transcriptase that participates in various human activities through telomere elongation and has enzyme activity in tumors (61). Past studies have confirmed that TERT is upregulated and altered in most cancer patients (62). Among STS, patients with TERT alternation have a shorter lifespan (63). TLR7 is a member of the Toll-like receptor family and plays a key role in the proliferation of tumor cells. It has been shown to be highly expressed in pancreatic cancer (64) and lung cancer (65). TRIM21 is a Fc receptor that can bind IgG, IgM and IgA (66). It has different expressions in different cancers, such as low expression in diffuse large B-cell lymphoma and breast cancer (67, 68), and high expression in human glioma and nasopharyngeal carcinoma cells (69, 70). Overexpression of TRIM21 makes osteosarcoma more resistant to various stresses and promotes its proliferation (71). In our study, TRIM21 expression was one of the reasons for poor STS prognosis. However, there are few studies on these 9 RBPs. In particular, it is difficult to find disease-related studies on MEX3A and SRSF12. Furthermore, we found that 9 RBPs had close interactions with transcription factors, microRNA and other RBPs. Previous research demonstrated that RBPs had similar patterns of somatic copy number analysis to transcription factors across 15 human cancer (72), which could support these findings. Therefore, these results might promote further research of RBPs in STS.

In addition, we compared the prognostic ability of these nine RBPs and clinical factors for STS. Univariate and multivariate Cox regression analyses showed that tumor metastasis and risk scores were independent prognostic factors. To accurately predict patient survival, we constructed a nomogram containing 9 RBPs. Previous nomograms have been developed to predict the OS of patients with STS based on tumor size, tumor grade, histological subtype and complete surgical resection (73). However, there are few nomograms for studying genes. Our study integrated the risk score and expression of 9 RBPs in the nomogram to further accurately predict the survival of patients with STS.

Subsequently, we further analyzed the changes of hub RBPs in STS and found that the alterations of key RBPs in STS patients are related to the shorter survival time, which further suggested that these 9 RBPs had high prognostic ability. In addition, we found that multiple methylation sites of upregulated genes were negatively correlated with their own expression in STS. These data showed that methylation changes may cause abnormal gene expression. The relationship between methylation of these RBPs and STS has rarely been studied before, and thus our study may provide a new direction for the treatment of STS.

Studies have shown that immune infiltration is an important factor affecting the prognosis of STS (74). We discussed the relationship between these 9 hub RBPs and immune infiltration. In this study, we found that most of these 9 hub RBPs have no correlation with tumor purity. Among them, TLR7 and TRIM21 were positively correlated with most immune cell infiltration, and MEX3A and SRSF12 were negatively correlated with most immune cell infiltration. DNMT3B was negatively correlated with CD4+ T cells, while TERT was positively correlated with CD4+ T cells. Some studies showed that TLR7 interacts with miR-25-3p, thereby stimulating the secretion of IL6 by macrophages and promoting the growth and spread of liposarcoma (75). Activation of TRIM21 expression induced infiltration of most immune cells except macrophages (76). IGF2BP3 was associated with immune cell infiltration in patients with liver cancer and affected prognosis (77). Activation of CD3EAP may cause T cells to produce IL2 to play an important role in non-small cell lung cancer (78). Therefore, the abnormal expression of these RBPs may alter the immune infiltrating components in STS, thereby achieving the purpose of treatment and improving the survival of patients.

Overall, based on the above series of analyses, the expression, participation mechanism and prognostic effects of the 9 hub RBPs were studied. These RBPs can be used as new biomarkers to predict the prognosis of STS and provide new ideas for the diagnosis and treatment of STS in the future. As far as we know, this was the first report to establish a prognostic model for RBPs related to STS. In short, our results will greatly facilitate the development of new diagnostic and therapeutic strategies and provide directions for future research.

## Data Availability Statement

The raw data were downloaded from TCGA and GEO database. Further inquiries can be directed to the corresponding authors.

## Author Contributions

L-LL, R-XW, Z-ZL and CH conceived and designed the study. R-XW, L-LL, Z-ZL, CH and XZ performed the analysis procedures. L-LL, MY, H-CZ, J-ZT, Z-ZL, WF, CH and XZ analyzed the results. L-LL, MY, CH and Z-ZL contributed analysis tools. J-ZT, L-LL, CH and R-XW contributed to the writing of the manuscript. L-LL, CH and R-XW performed project administration. All authors contributed to the article and approved the submitted version.

## Conflict of Interest

The authors declare that the research was conducted in the absence of any commercial or financial relationships that could be construed as a potential conflict of interest.

## Supplementary Material

Supplementary Figure 1 Association of methylation sites with expression of STS hub RBPs. Note: **(A)** BYSL, **(B)** CD3EAP, and **(C)** MEX3A.

Supplementary Figure 2 Potential interactions of 9 hub RBPs. Note: **(A)** TLR7, **(B)** TERT, **(C)** SRSF12, **(D)** MEX3A, **(E)** IGF2BP3, **(F)** DNMY3B, **(G)** CD3EAP, **(H)** BYSL and **(I)** TRIM21.

Supplementary File 1 Detailed information of patients with STS.

Supplementary File 2 The expression matrix of 1492 RBPs from STS and normal tissue samples.

Supplementary File 3 Detailed information of 329 RBPs. Note: 272 shared genes between the PPI network and 329 differentially expressed RBPs were marked with red color.

Supplementary File 4 The interactions of nodes in PPI network.

Supplementary File 5 The centrality indexes of nodes in PPI network.

Supplementary File 6 Detailed information of identification of subnetworks using MCODE app.
