# Supplementary figures and images for: Integrated Analysis of Nine Prognostic RNA-Binding Proteins in Soft Tissue Sarcoma
Source: Front Oncol. 2021 May 7;11:633024. doi: 10.3389/fonc.2021.633024 (PMC8138553; doi:10.3389/fonc.2021.633024)

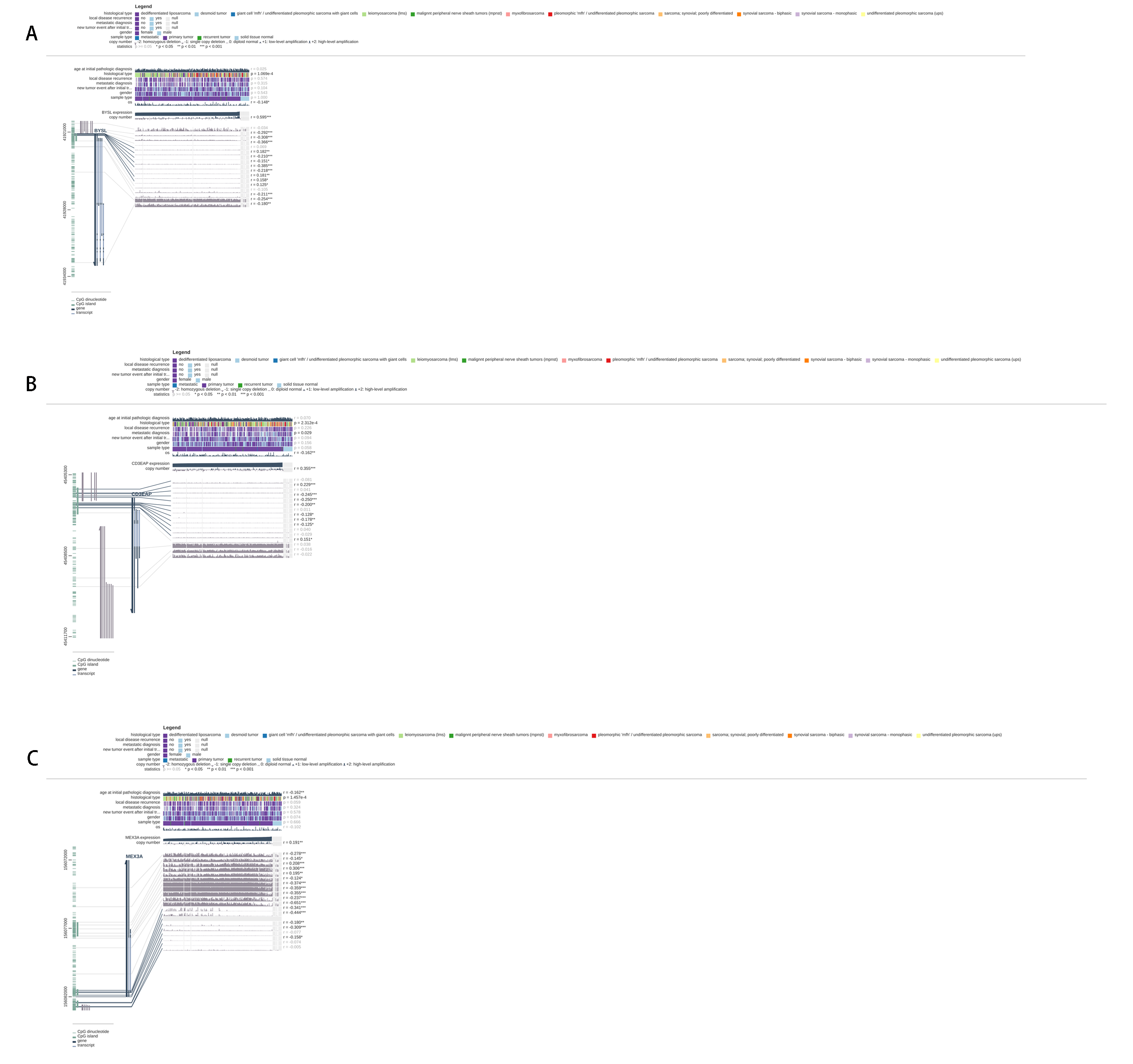

Supplement: Supplementary Figure 1 — Association of methylation sites with expression of STS hub RBPs. Note: (A) BYSL, (B) CD3EAP, and (C) MEX3A. [file DataSheet_1.zip › Supplementary Materials/Supplementary Figure 1.tif]

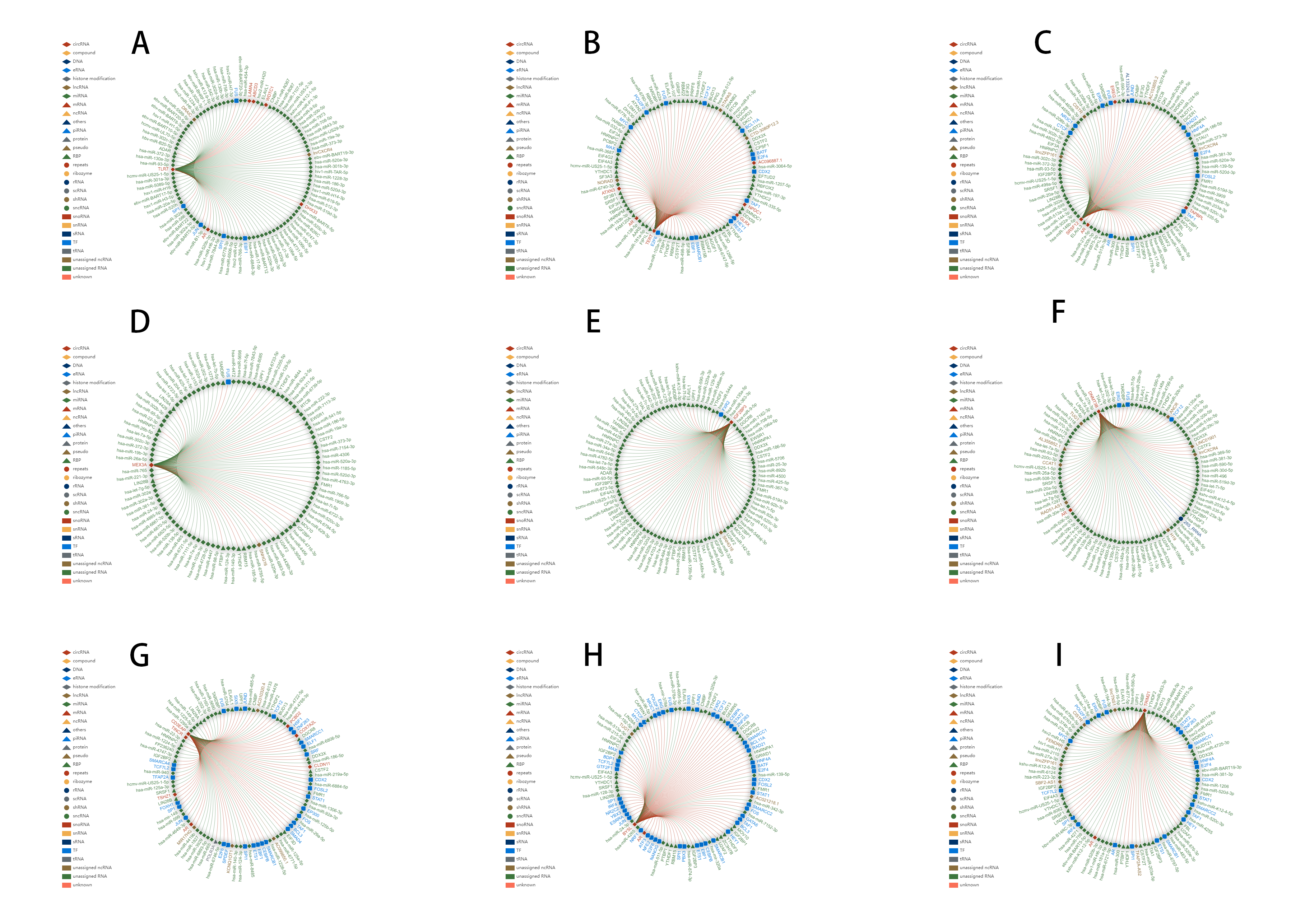

Supplement: Supplementary Figure 1 — Association of methylation sites with expression of STS hub RBPs. Note: (A) BYSL, (B) CD3EAP, and (C) MEX3A. [file DataSheet_1.zip › Supplementary Materials/Supplementary Figure 2.tif]
